# Perceived risk and attitude's mediating role between tourism knowledge and visit intention during the COVID-19 pandemic: implementation for coastal-ecotourism management

**DOI:** 10.1016/j.heliyon.2022.e10724

**Published:** 2022-09-23

**Authors:** Zainal Abidin, Wahyu Handayani, Emeraldo A. Zaky, Achmad D. Faturrahman

**Affiliations:** a Fisheries Socioeconomics (PSDKU) Study Program, Universitas Brawijaya, Indonesia; b Fisheries Agribusiness Study Program, Universitas Brawijaya, Indonesia

**Keywords:** Coastal ecosystem management strategy, Ecotourism, TRA, Visit intention, COVID-19

## Abstract

CMC Tiga Warna is one of the coastal area management for conservation and ecotourism in southern Malang, Indonesia. Despite fewer tourist visits, the COVID-19 pandemic did not dissuade locals from managing and protecting ecotourism in the coastal area. This study aims to: (a) to describe coastal ecosystem management; and (b) to analyze coastal ecotourist visit intention related to tourism knowledge, perceived health risk, and risk attitude in the post-COVID-19 era as a part of developing strategy for coastal ecosystem management in the study area from the perspective of ecotourists and destination management. CMC Tiga Warna management, community group supervisors, the local community, and a variety of experts were interviewed in order to gather data for the current strategy. Coastal ecotourist visit intention data from domestic ecotourists was gathered via online and offline surveys. Analyzing the visit intention data with Warp-PLS. Using the visit intention model and interview data, the coastline management strategy was described. This study found that the coastal environment management plan constantly involved ecotourists and locals. Tourism knowledge and risk attitude are positively related to the visit intention, however perceived health risk is negatively related to it. The significant positive relationship between tourism knowledge and ecotourist visit intention is mediated by perceived health risk and risk attitude. Development of the coastal ecosystem management plan could be supported by boosting visit intention, ecotourism visits, or economic incentive as a motive for sustaining the conservation program's consistency, and ecological and social rewards in a sustainable manner. This study added to the Theory of Reasoned Action by adding two additional factors, tourist knowledge and perceived health risk, in addition to the attitude to predict the visit intention. The ecotourism manager should provide varied tourist information and knowledge as needed, reduce COVID-19 exposure risk at tourist destinations, and increase risk attitude.

## 1 Introduction

Clungup Mangrove Conservation (CMC) Tiga Warna has become one of Malang Regency's coastal ecotourism sites, as well as one of Indonesia's coastal places that regularly maintains mangrove and coral reef conservation. Ecotourism management has consistently prioritized ecological protection above the use of limited natural resources for ecotourism (Abidin et al., 2021). The increasing number of tourist visits in 2019 was viewed as more aggressive. However, there was a drop in visits during the pandemic of COVID-19. The local community's readiness to implement health procedures in CMC Tiga Warna tourist area has begun to encourage tourist’ intent to return. However, the disparity in the amount and quality of public knowledge about contemporary tourist circumstances connected with the COVID-19 exposure risk and the execution of health protocols has prevented CMC Tiga Warna from fully recovering. It is also more timely due of the ongoing COVID-19 Pandemic in Indonesia. It has evolved into a global public health concern (Zhu and Deng, 2020). Tourism, according to Neuburger and Egger (2020), Roman et al., (2020) and Niewiadomski (2020), has been the hardest damaged and interrupted by the Covid-19 outbreak. Thus, understanding the feasibility of implementing the coastal ecotourist visit intention model in the post-COVID-19 period as a supporting component for building the management of coastal ecosystem strategy from the perspective of tourism market and management is deemed critical.

The theory of consumer behavior has been referred to predict behavioral intents and consumer decisions (Steinbauer and Werthner, 2007), which are thought to be influenced in a tourist viewpoint by tourism knowledge connected to health risk (Zhu and Deng, 2020), such as during the COVID-19 pandemic (Zhu and Deng, 2020; Utama and Setiawan, 2020). Health risk perceptions vary based on one's background, and if not properly designed, monitoring programs might become discriminatory; consequently, understanding them is essential (Williams et al., 2022). Furthermore, the Theory of Planned Behavior (TPB), a modification of the Theory of Reasoned Action (TRA), has been frequently used to explain tourist behavior and predict whether someone will come. TPB employs three drivers of behavioral intention: attitudes, subjective norms, and control of perceived behavior (Ajzen, 1991). Attitude is an overall examination of personal behavior that examines the decision to visit a tourism destination. Subjective norms are a person's view based on the thoughts of key individuals in his life, which influences whether or not he performs a behavior. View of behavioral control is a person's perception of whether it is acceptable to carry out an activity, which is impacted by individual beliefs about the availability and role of resources (Ajzen, 2015). Behavioral intention is thus a person's tendency to do or not do a particular task (Ajzen, 1991).

**Figure 1:**
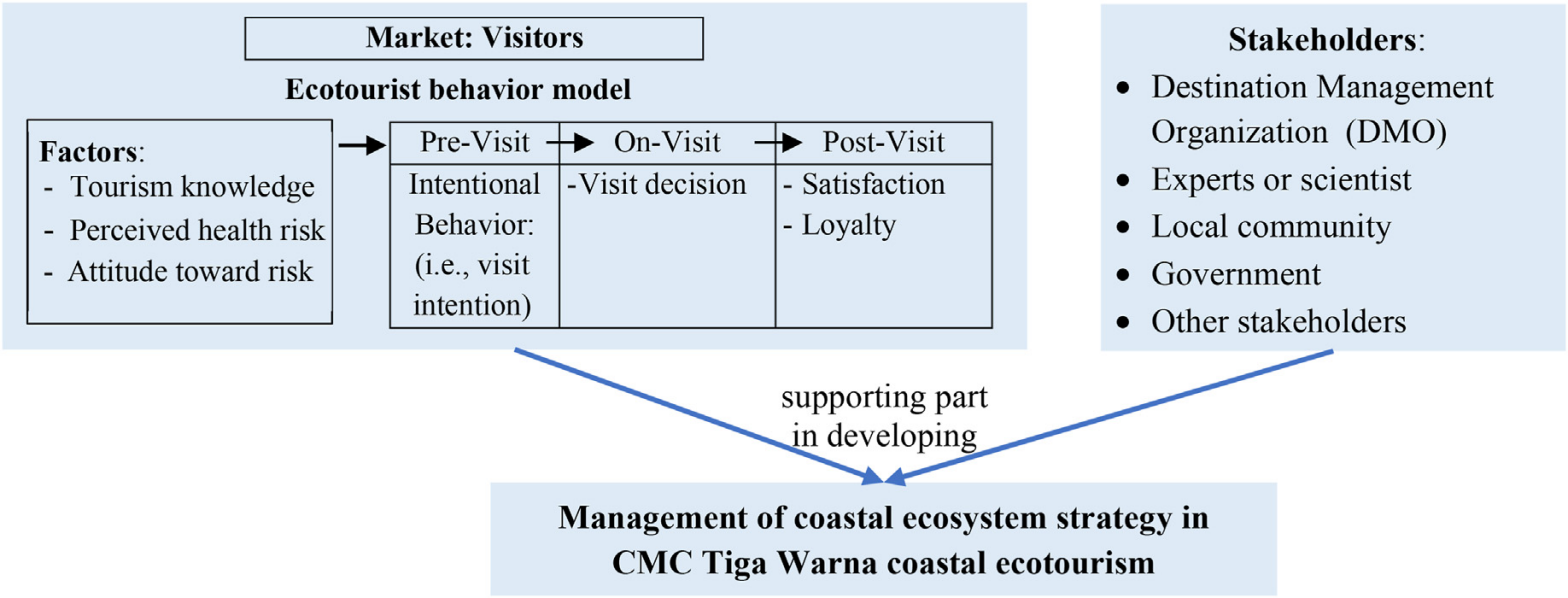
Conceptual framework.

Empirical research regarding the tourist behavior with TRA and TPB approaches has been extensive; however, specific research expanding the application of TRA and TPB in coastal ecotourism during post-COVID-19 by adding the tourism knowledge factors and the COVID-19 exposure risk has been limited, particularly in developing countries such as Indonesia. Furthermore, empirical research that utilizes tourist behavior studies to develop the management of coastal ecosystem strategy has been insufficient. Currently, the majority of studies have usually linked behavioral intention with the TPB approach (Jalilvand and Samiei, 2012; Chen and Tung, 2014; Isa and Ramli, 2014; Abubakar and Ilkan, 2016; Cong, 2016; Doosti et al., 2016; Wang et al., 2018; Bashir et al., 2019; Kusumawati et al., 2020). There has been no empirical research on specific behavioral intentions in coastal ecotourism to promote interest in ecotourism visits in the post-COVID-19 period and to support the development of the coastal ecosystem management strategy. Another study however focuses on the interest in visiting rural tourism during the COVID-19 pandemic (Zhu and Deng, 2020), the travel interest associated with the exposure risk (Utama and Setiawan, 2020), and the intention to return during the COVID-19 pandemic (Hassan and Soliman, 2021). As a result, the purpose of this study is to broaden the use of TRA and TPB in coastal ecotourism in order to improve the prediction ability of behavioral intention (Ajzen, 1991), as well as to further develop the coastal ecosystem management strategy from perspective of the tourism market and management. Thus far, this research aims to: (a) describe coastal ecosystem management in CMC Tiga Warna, and (b) analyze coastal ecotourist visit intention in relation to tourism knowledge, perceived health risk, and attitude toward risk in the post-COVID-19 phase to aid in the development of the coastal ecosystem management strategy in the study area. Figure 1 depicts the conceptual framework.

## 2 Literature review and hypothesis development

This study makes use of a variety of concepts and theories, including consumer behavior theory, Theory of Planned Behavior (TPB), tourism knowledge (connected to risk), and perceptions of health risks (the risk of exposure to Covid-19), and ecosystem management. The study of theory and the concept of consumer behavior is still evolving. The primary consumer behavioral opinion (Engel et al., 1968; Hawkins et al., 2004; Dwiastuti et al., 2012) that consumer behavior is divided into three stages: before, during, and after the purchase, and is influenced by both internal and external variables.

Consumer behavior theory is being created in order to better understand and explain customer decisions and behavior, as well as to get practical implications for predicting consumer intentions and behaviors (Steinbauer and Werthner, 2007), which, in the context of tourism, is also influenced by tourism knowledge related to risk (Zhu and Deng, 2020) and perceived health risk similar to the Pandemic Covid-19 era.

TPB is a revision of theory of Reasoned Action (TRA). Ajzen and Fishbein expanded TRA through human behavior studies. In 1988, behavioral control perception was added to TRA and named TPB (Ajzen, 1991). TPB is used to predict whether someone does or does not do a behavior. TPB uses three intentional decisive constructs, namely attitude, subjective norms, and behavioral control perceptions (Ajzen, 1991).

Attitude is an assessment of one's general personal behavior as well as a decision to visit a tourism site. The indicators are as follows: wise, good concept, and support. Subjective norms are defined as a person's view based on the thoughts of key individuals in his life, as to whether or not he did or did not conduct a behavior. Advice, influence, and approbation are the indicators. Perceived behavioral control is a person's perception of how simple or difficult it is to realize a behavior, which is impacted by individual perceptions about the availability and role of resources in behavior realization (Ajzen, 2015). The indicators include freedom, resources, and thus will. The intention behavior is a person's proclivity to do or not do a job. According to Ajzen and Fishbein (1980), purchase intentions are described as customers' proclivity to own specific things, and they have been shown to be a dominant predictor of consumer behavior. Possibilities, wishes, intents, and plans to visit are the indicators. The perception of destination risk can be changed by previous travel and visits (as knowledge), and then the perception of destination risk is the driver of the intention to travel to Host City (Schroeder et al., 2013). During the Pandemic Covid-19 period, the sense of destination risk is also related to the perception of danger of exposure to Covid-19 while visiting a tourist location. According to LEPP and Gibson (2003), tourists with a wide range of tourism experiences and sufficient risk understanding perceive lower hazards. It is also consistent with the discovery by Zhu and Deng (2020) that tourism knowledge related to risk has been shown to have a detrimental effect on risk perceptions. Attitudes toward hazards and perceptions of risks are equally effective in mediating the impact of tourism knowledge on visit intention. As a result, the hypotheses are stated as follows:

H1 There is a negative relationship between tourism knowledge and perceived health risk.

H3 There is a positive relationship between attitude toward risk and coastal ecotourist visit intention.

H4 Perceived health risk and attitude toward risk mediate the significant positive relationship between tourism knowledge and coastal ecotourist visit intention.

H5 There is a positive relationship between tourism knowledge and coastal ecotourist visit intention.

Consumers incur risk when they are unable to forecast the repercussions of their purchase actions. Risk perception is defined as a subjective estimate of a person's concern about the probability of a goods-related accident, as well as how concerned they are about the consequences or impacts of the incident (Wahyuningtyas, 2015). Risk perceptions in tourism are described as an individual's views of the possibility of an activity that can explain a threat and impact travel decisions (Chew and Jahari, 2014). Tourists are concerned about risk because they want to feel comfortable and secure about their travel plans. Previous studies broaden the topic of study by investigating the relationship between prior tourist knowledge, risk perceptions, and information seeking behavior (Sharifpour et al., 2013).

Because the trip is not the primary objective, travel and tourism are more prone to risk and uncertainty. Tourists are also more risk averse (Artuğer, 2015). Additionally, it is more pertinent at this time due of the ongoing Pandemic Covid-19 in Indonesia. The Covid-19 outbreak has developed into a global public health emergency (Zhu and Deng, 2020). Individuals and society may be harmed as a result of the extensive effects. According to Neuburger and Egger (2020), tourism is the sector that has been hardest hit by the Covid-19 outbreak. Vulnerability is a term that relates to the possibility of developing a disease (Brewer and Fazekas, 2007).

Health risk can be described as a subjective risk of adverse health events occurring to an individual or a group of individuals during a specified time period. Study by Zhu and Deng (2020) proving that tourist visit intentions are negatively impacted by the risk perception of the Covid-19. Other researcher Schroeder et al., (2013), there was also evidence that the risk perception of the location was a driver of the intention to travel to the host city, as long as demographic parameters and previous experiences were controlled. The second hypothesis is as follows:

H2 There is a negative relationship between perceived health risk and coastal ecotourist visit intention.

Ecosystem management arose from the need for a long-term strategy to understanding the intricate interrelationships that exist between various, and sometimes competing, aspects of the environment as they work together to produce ecosystem services. So, integrating the ecological, economic as well as social dimensions of ecosystem services is important to be a reality to make sustainable coastal ecosystem management such as seen in Caribbean Small Island Developing States (SIDS) by making a collaborative effort between decision-makers and scientists (Ali et al., 2018). Stakeholder collaboration as a major factor for sustainable ecotourism development in developing countries (Wondirad et al., 2020). Management of coastal ecosystem in a number of Indonesian locations, including mangrove ecosystem management in Lombok (Sukuryadi et al., 2021), and in the Special Region of Yogyakarta (Khakhim et al., 2021), management of coastal biodiversity in Gorontalo Province (Katili et al., 2018) and coastal management using a Community-Based Tourism (CBT) model in Panggul, Trenggalek of East Java Province (Sarinastiti and Wicaksono, 2021).

## 3 Materials and methods

### 3.1 Study sites

**Figure 2:**
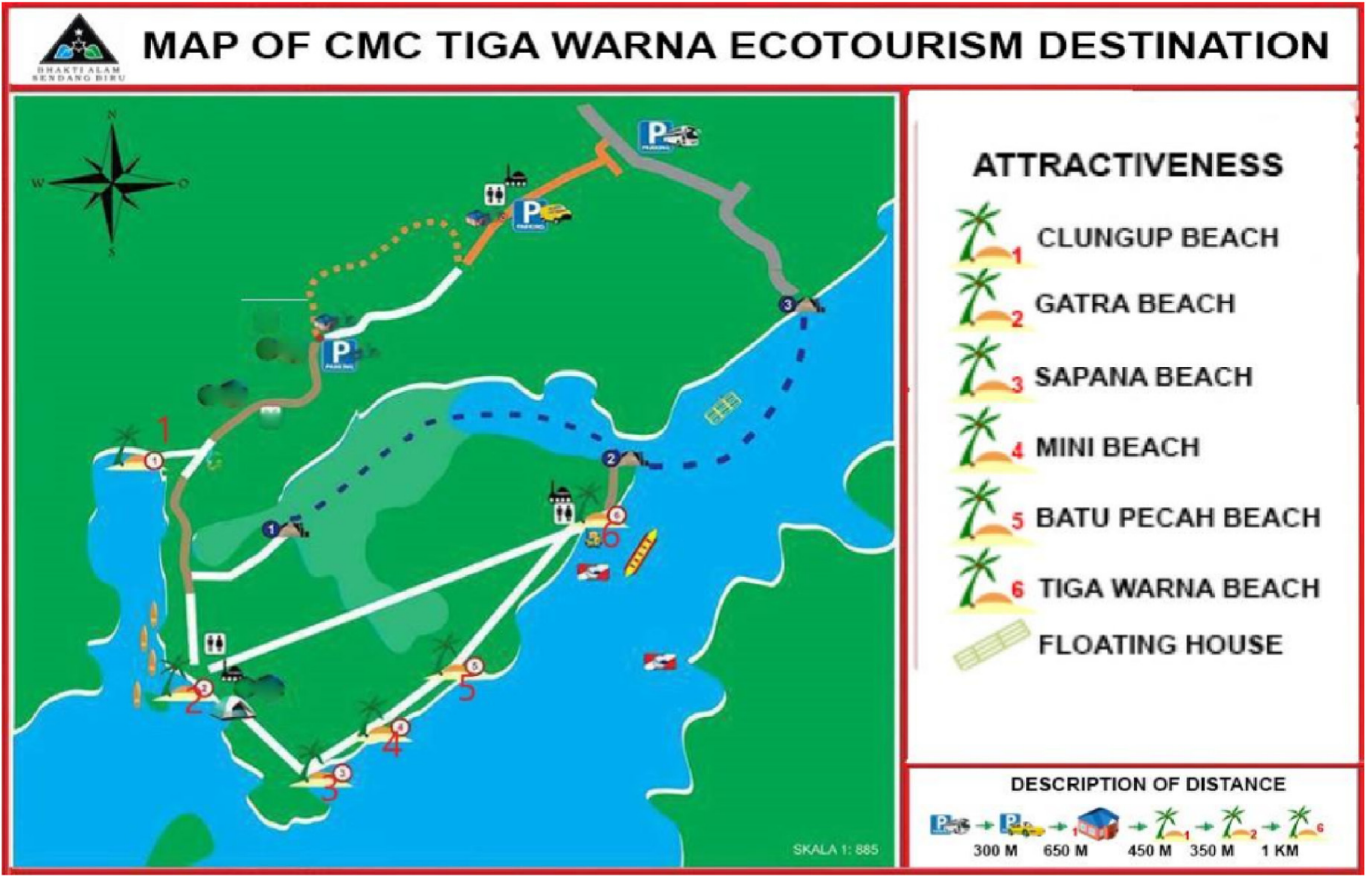
Map of research location: CMC Tiga Warna coastal ecotourism, southern Malang. (Source: Riniwati et al., 2019).

This research took place in Malang's southern region, specifically in the coastal area which is managed as a coastal protected area and ecotourism destination with the brand entitled CMC Tiga Warna, acknowledged for its consistency in restoring coastal ecosystems, especially devoted for mangroves and coral reefs through conservation strategies. Research location map is illustrated in Figure 2.

### 3.2 Procedures

This study employs a mixed methodological approach, which includes both quantitative and qualitative methods (Creswell, 2016). Explanatory research using a survey of coastal ecotourist visit intention for the quantitative method. Interviews related to the present management of coastal ecosystem strategy with CMC Tiga Warna administrators, tour guides, community group supervisors, and local communities were used to conduct the qualitative technique. Data was additionally obtained not only by implementing interviews method, but also observations, and documentation activities regarding the condition of coastal ecosystem management in CMC Tiga Warna. Meanwhile, from May to June 2021, data regarding the coastal ecotourist visit intention from domestic ecotourists were gathered both online and offline surveys utilizing Likert scale questionnaires with a score of 1–5 (strongly disagree to strongly agree), by filtering the respondents through several pre-questions to fulfill the sample requirements, including 17-year-old domestic tourists who visited the CMC Tiga Warna for at least one time from 2019 to 2020. Respondents had understood and approved to fill out the questionnaire for data collecting and publication purposes. They did it after reading the introduction in the beginning of interviewing. So, the informed consent was obtained from all participants. Based on the sampling technique used for Warp-PLS analysis at least 100 respondents (Solimun et al., 2017), finally a total of 315 and 47 respondents were separately obtained from online and offline questionnaires, generating the 348 qualified respondents (96.1%) after discarding the incomplete responses to be further analyzed.

Based on the profile of CMC Tiga Warna ecotourism that the previous visitors population in the study area were dominated by young persons (YBAS, 2020). It was also relevance with the demographic characters of respondents (Table 1) where their average age was between 17 to 25 years old (78%), included female (64%) and male (36%). Most of them are unmarried marital status (85%) and come from East Java Provinces areas such as Malang City, Surabaya, Malang Regency, Sidoarjo, and Mojokerto (83%). They have relatively high level education or graduated from high education (56%), with the most common activity being of a student (44%) and private employees (29%). Most of their monthly income (pocket money) of < IDR 2,500,000 (59.5%) and IDR 2,500,000 to 4,000,000 (21.8%); and monthly spending of < IDR 2,500,000 (65%) and IDR 2,500,000–4,000,000 (23%). Lastly, most of them with once or twice visit frequency (75%), and searching intention for travel information from friend or family (49%%) and social media (43%).

**Table 1:** Respondent characteristics.

Respondent characteristics		Amount (Person) and Percentage (%)
Sex	Male	125 (36.0%)
	Female	223 (64.0%)
Place of Origin	East Java province	288 (83.0%)∗)
	Outside East Java province	60 (17.0%)
Age (year)	17–25	271 (78.0%)
	26–36	61 (17.5%)
	36–45	11 (3.2%)
	>45	5 (1.4%)
Marital status	Single	295 (85%)
	Married with no children	24 (6.9%)
	Married with children	29 (8.3%)
Education level	Primary education (SD/MI)	33 (9%)
	Secondary education (SMP/MTS)	4 (1.0%)
	Secondary education (SMA/SMK/MA)	116 (33%)
	Degree (Diploma, S1, S2, S3)	195 (56%)
Occupation	Student	154 (44%)
	Civil servant	6 (1.7%)
	Private employee	102 (29%)
	Entrepreneur	37 (11%)
	Housewife	10 (2.9%)
	Household personal assistant	9 (2.3%)
	Unemployed	30 (8.6%)
Religion	Moslem	328 (94%)
	Christian and Catholic	20 (6%)
Income	< IDR 2,500,000	207 (59.5%)
	IDR 2,500,000–4,000,000	76 (21.8%)
	>IDR 4,000,000	65 (18.7%)
Frequency of visits	1-2 times	261 (75%)
	>2 times	28 (8.0%)
Source of information	Family/friends	170 (49.0%)
	Social Media	149 (43.0%)
	Others	29 (8.3%)

∗) 56% from areas such as Malang City, Surabaya, Malang Regency, Sidoarjo, and Mojokerto, which are all within 80–165 km of the CMC Tiga Warna.

Since Likert scale data are ordinal, they should not be used to calculate means or standard deviations at the individual item level (Sullivan and Artino, 2013). Using the Warp-PLS tool, Likert-scale data was standardized in the form of Z data during the PLS-SEM analysis stages, so that the mean = 0, and standard deviation = 1 (Solimun et al., 2017).

The measurement of tourism knowledge applied the indicators adopted from Zhu and Deng (2020), including: the information regarding tourism, the causes of tourism risks, the consequences of tourism risks, and the solutions to tourism risks. Perceived health risk variable was measured based on indicators adapted from Zhu and Deng (2020); Utama and Setiawan (2020), which included: the contracting risk of COVID-19 due to travel, the contracting risk of infection even though the tourist location has implemented the visit restrictions, the worrisome about the spread of COVID-19 in southern Malang area, the travel refraining, and the worrisome about the handling of health at tourist sites. Furthermore, the measurement of the attitude toward risk variables was performed by using indicators which were: thoughtful, calculable, and supportive. Meanwhile, coastal ecotourist visit intention measurement was performed by using 4 indicators which included: possibilites, desires, intentions, and plans to visit (Ajzen and Fishbein, 1980).

### 3.3 Data analysis

Data regarding to the condition of coastal ecosystem management and its strategy were studied using qualitative descriptive analysis. Furthermore, the Warp-PLS approach was used to examine the visit intention in the post-pandemic COVID-19 era as influenced by tourism knowledge, perceived health risk, and attitude toward risk. The use of PLS-SEM with the Warp-PLS application is also relevant to analyze the direction and correlational relationships (Pesämaa et al., 2021). Since this study focuses on theory development as well as prediction purposes, PLS-SEM is preferable to CB-SEM (Hair et al., 2017). Warp-PLS can work with small or large samples. Besides, Warp-PLS does not require a strong basic theory, such as SEM (Solimun et al., 2017). For this reason, Warp-PLS is probably more appropriate for theory development. Measurement and structural modeling were carried out in this case. The measurement model comprises of questionnaire validity and reliability tests (Table 2), while the structural model employs the Goodness of fit test for the proposed study model (Table 3). Following the completion of confirmatory factor analysis (CFA), these tests were assessed. All validity and reliability criteria were acceptable and reliable. The resulting structural model met all of the criteria.

**Table 2:** Rule and result of validity and reliability test.

Test	Parameter	Rule of Thumb	Result
Validity	Factor loading value	>0.3 is considered acceptable	Valid
	Average Variance Extracted (AVE)	>0.5 is considered acceptable. However, if < 0.5 does not cause a concern, it is acceptable	Valid
Reliability	Composite reliability coefficients	>0.7 is considered reliable	Reliable
	Cronbach's alpha coefficients	>0.6 is considered reliable	Reliable

**Table 3:** Model fit and quality indices.

Model Fit and Quality Indices	Fit Criterion	Analysis Result	Remark
Average path coefficient (APC)	P < 0.05	0.244 (P < 0.001)	Good
Average R-squared (ARS)	P < 0.05	0.131 (P = 0.003)	Good
Average adjusted R-squared (AARS)	P < 0.05	0.127 (P = 0.004)	Good
Average block VIF (AVIF)	Acceptable if ≤ 5, ideally ≤3.3	1.130	Ideal
Average full collinearity VIF (AFVIF)	Acceptable if ≤ 5, ideally ≤3.3	1.188	Ideal
Tenenhaus GoF (GoF)	Small ≥0.1, medium ≥0.25, large ≥0.36	0.317	Medium
Sympson's paradox ratio (SPR)	Acceptable if ≥ 0.7, ideally = 1	1.000	Ideal
R-squared contribution ratio (RSCR)	Acceptable if ≥ 0.9, ideally = 1	1.000	Ideal
Statistical suppression ratio (SSR)	Acceptable if ≥ 0.7	1.000	Acceptable
Nonlinear bivariate causality direction ratio (NLBCDR)	Acceptable if ≥ 0.7	1.000	Acceptable

## 4 Results

### 4.1 Management and utilization of coastal ecosystems in CMC Tiga Warna ecotourism

CMC Tiga Warna is acknowledged as an ecotourism destination managed by local communities who are members of the Bhakti Alam Sendang Biru Foundation (YBAS). This foundation has managed coastal ecosystems focusing on rehabilitation and conservation, particularly devoted for mangrove and coral reef ecosystems. Additionally, the two other ecosystems are conserved, which include: the coastal border and the seagrass, similar to the management of coastal ecosystems in other areas in Indonesia, such as in Gorontalo Province consisting of three main components of managed coastal ecosystems, including: coral reef, mangrove and seagrass (Katili et al., 2018). Whereas in Panggul District of Trenggalek Regency, there are mangrove ecosystems and other coastal biodiversity managed for conservation and ecotourism purposes. Ecotourism refers to a form of limited use of the conserved coastal ecosystem to generate economic incentives as a complement to the ecological incentives, created through conservation. Hence, the economic incentives in this study are positioned as financial capital to encourage local communities in conducting the conservation on an ongoing basis. According to another study conducted by Harahab et al. (2021), management of marine ecotourism in CMC Tiga Warna was perceived to be highly sustainable, particularly in terms of conservation, in contrast to management of mangrove ecosystem in Lembar Bay, Lombok, Indonesia, which Sukuryadi et al. (2020) described as less-sustainable. On this basis, the management and utilization of coastal ecosystems in CMC Tiga Warna are required to be consistent in sustaining the conservation programs engaging both visitors and local communities. Through this research, the coastal ecotourist visit intention as a factor originating from the tourism market has been perceived as a novelty, serving as a supporting part for developing strategy of coastal ecotourism management in CMC Tiga Warna.

### 4.2 Conservation policy and zoning of coastal ecotourism destinations CMC Tiga Warna ecotourism

Previous study Abidin et al., (2020) reported that coastal biodiversity conservation strategy has been focused on maintaining a balance between nature's ability and its restricted use through ecotourism as part of the coastal ecosystem performed in CMC Tiga Warna ecotourism. It applies the two ecotourism destination zonings of: (1) emission-free areas, consisting of the application of pedestrian paths, and the application of motorized vehicle lanes; and (2) limited use for ecotourism in mangrove conservation area and coral reef protected areas.

Utilization of coastal ecosystems for tourism purposes has however strived to provide continuous opportunities for nature to recover, thereby encouraging the management activities at CMC Tiga Warna ecotourism which include: a). the capacity implementation of coastal ecotourism zoning, by restricting the visitors to the conservation area in the three beaches (Tiga Warna, Clungup, and Gatra) in order to preserve nature; b). the procurement and development of tourism facilities and infrastructures to sustain the environmental carrying capacity; and c). the development of ecotourism which leads to a healthy tourism business. Thus far, CMC Tiga Warna ecotourism has improved benefitting economically for local communities, despite currently focusing on the environmental sustainability.

### 4.3 Coastal ecosystem management achievement in CMC Tiga Warna ecotourism

According to YBAS (2020), ecological achievements from the successfully implementation programs regarding conservation management activities in CMC Tiga Warna area, include: the release of 27 ha of coastal forest independently from 96.24 ha, which should be a green belt, the release of 6 ponds to become a mangrove rehabilitation and conservation area with a total area of 74 ha of mangroves, the release of 59 ha of Marine Protect Area from previously 5 ha out of 14.4 ha, the release of the 37 identified species of mangrove species, including major mangroves (17 species), minor mangroves (6 species), combined mangroves (14 species), along with various animals such as long-tail monkeys, squirrels, otters, hedgehogs, monitor lizards, snakes, birds, and others.

The implementation of conservation in CMC Tiga Warna coastal ecotourism has proven to be effective in maintaining sustainability and environmental awareness (Abidin et al., 2021) as it is consistent in applying the principles of conservation, currently managed by involving visitors and local communities. However, the questioned managerial strategy lies in the implementation of ecotourism management in CMC Tiga Warna. The strategy of involving the community as managers in every conservation activity has been intended for local communities to receive economic incentives towards their role in conserving the coastal ecosystems. Attempts regarding the visitor involvement strategy have been initiated from reservations, point of entry and exit in ecotourism areas through checking luggage that has the potential for waste, and others in order to educate the public and visitors as well as to increase the environmental awareness.

### 4.4 Result of Warp-PLS: coastal ecotourist visit intention model in the post-COVID-19 period

#### 4.4.1 Measurement model

Confirmatory Factor Analysis (CFA) was used to examine the questionnaire's reliability and validity, and the results indicated that all of the factor models fit the data and that the loading factors criteria were achieved. Table 4 depicts that the convergent test also fulfilled the criteria. In addition, discriminant validity was attained. Furthermore, the questionnaire's composite reliability and Cronbach's alpha met the standards, indicating that it was both reliable and valid.

**Table 4:** Validity and reliability of questionnaire.

Variables, indicators, and items		Factor loading	Sq. roots AVE	Composite reliability	Cronbach's alpha
Tourism knowledge			0.848	0.910	0.866
X1.1	Information regarding travel	0.698∗∗∗			
X1.2	Causes of tourism risk	0.908∗∗∗			
X1.3	Consequences of tourism risk	0.924∗∗∗			
X1.4	Solutions for travel risk	0.845∗∗∗			
Perceived health risk			0.885	0.947	0.930
X2.1.1	The contracting risk of the COVID-19 due to travel	0.913∗∗∗			
X2.1.2	The contracting risk of the COVID-19 even though the tourist location has implemented restrictions on visits	0.933∗∗∗			
X2.2	There is a spread of the COVID-19 in southern Malang	0.908∗∗∗			
X2.3	The travel refraining	0.848∗∗∗			
X2.4	The worrisome concerning health care at tourist sites	0.817∗∗∗			
Attitude toward risk			0.862	0.920	0.884
X3.1.2	Wise actions to comply with the health protocols	0.898∗∗∗			
X3.2	Supportive idea to comply with the health protocols	0.918∗∗∗			
X3.3	Supportive travel decisions	0.840∗∗∗			
X3.4.1	Cautious actions to comply with health protocols	0.788∗∗∗			
Coastal ecotourist visit intention			0.904	0.947	0.926
Y.1	Possibility of traveling	0.893∗∗∗			
Y.2	Desire to travel	0.903∗∗∗			
Y.3	Travel intentions	0.931∗∗∗			
Y.4	Travel plans	0.890∗∗∗			

Notes: ∗∗∗p < 0.001.

#### 4.4.2 Structural model

**Figure 3:**
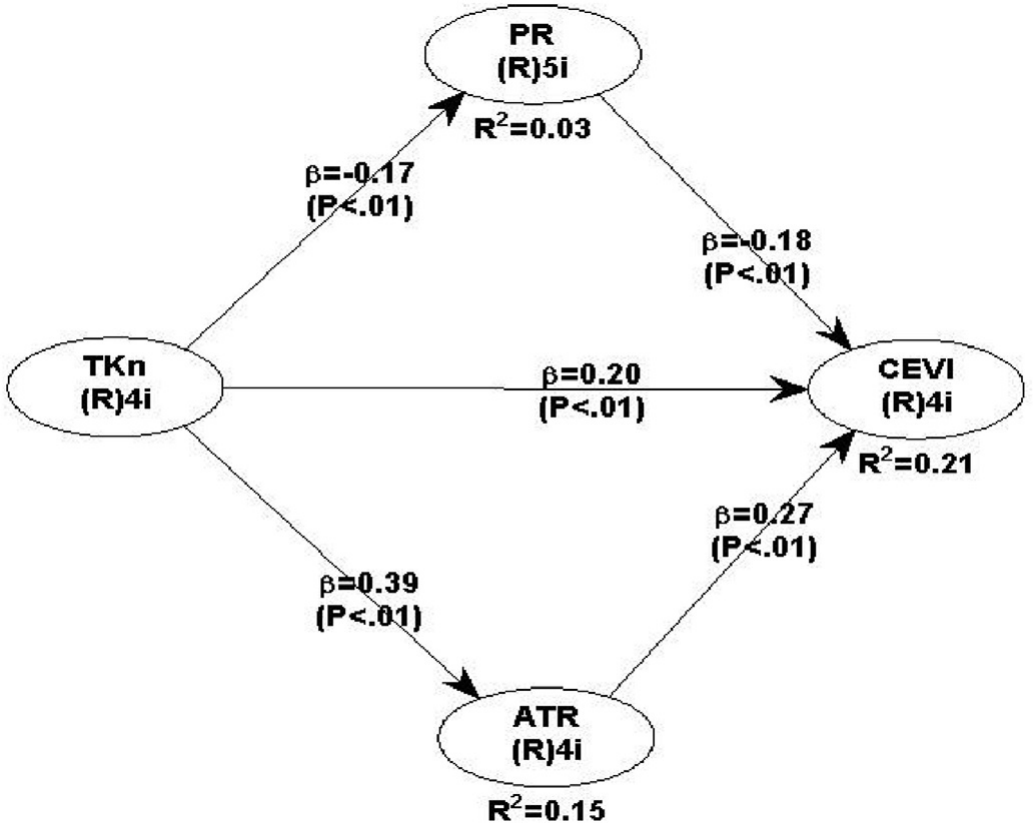
Structural model resulted. Note: ∗∗∗ Significant at 0.01 level.

The model fit test (Table 3) was used to evaluate the suggested model. The p-value for the three main quality indices is significant at 0.01 for APC = 0.244, ARS = 0.131, and AARS = 0.127. Further, the proposed model is explained by variance percentages of 3, 15, and 21 in perceived health risk, attitude toward risk, and coastal ecotourist visit intention, respectively. The resulting model has a high fit value and could be used to forecast the development of coastal ecotourist visit intention during post COVID-19. The hypotheses were tested by performing the t test with 1% alpha level. Table 5 indicates a negative relationship between tourism knowledge and perceived health risk at the 0.01 significant level. Perceived health risk, on the other hand, was adversely and substantially related to the coastal ecotourist visit intention at the 0.01 significant level, as did tourism knowledge. At the 0.01 significant level, the indirect effect of tourism knowledge on coastal ecotourist visit intention via perceived health risk and attitude toward risk was similarly substantial. In conclusion, all hypotheses are accepted, with the results given in Table 5. While the coastal ecotourist visit intention model resulted shown in Figure 3.

**Table 5:** Hypotheses testing.

Hypotheses number	Paths	Path coefficient	Supported
1	TKn → PR	−0.171∗∗∗	Yes
2	PR → CEVI	−0.182∗∗∗	Yes
3	ATR → CEVI	0.269∗∗∗	Yes
4	TKn → PR & ATR → CEVI	0.137∗∗∗	Yes
5	TKn → CEVI	0.205∗∗∗	Yes

Notes: TKn: Tourism knowledge, PR: Perceived health risk, ATR: Attitude toward risk, CEVI: Coastal ecotourist visit intention, ∗∗∗ Significant at 0.01 levels.

#### 4.4.3 The best path to determine visit intention in the post-COVID-19 phase

The results of both direct and indirect hypothesis testing indicate the degree of effectiveness of each path for increasing visitor numbers. Table 6 demonstrates this as well.

**Table 6:** Effectiveness of all paths.

Variables			Type of Effect			Priority of the effectiveness	
			Direct Effect (DE)	Indirect Effects (IE)	Total Effect (TE)		
P	M	R	β (p-value)	(p-value)	(p-value)	All paths to VI (DE & IE)	DE to CEVI
TKn	-	PR	−0.171 (<0.001)	-	−0.171 (<0.001)	- -	
TKn	-	ATR	0.392 (<0.001)	-	0.392 (<0.001)	- -	
TKn	PR	CEVI	-	0.137 (0.005)	0.342 < 0.001	1	-
	ATR		-				-
	-		0.205 (<0.001)	-			2
PR	-	CEVI	−0.182 (<0.001)	-	−0.182 (<0.001)	-	-
ATR	-	CEVI	0.269 (<0.001)	-	0.269 (<0.001)	2	1

Notes: P: Predictor variables; M: Mediating variables; R: Response variables; TKn: Tourism knowledge; PR: Perceived health risk; ATR: Attitude toward risk; and CEVI: Coastal ecotourist visit intention.

## 5 Discussion

Discussion regarding the development of coastal ecosystem management strategy in CMC Tiga Warna in the post-COVID-19 era is described by utilizing the results of behavioral analysis of interest in visiting CMC Tiga Warna ecotourism. Analysis of the coastal ecotourist visit intention in the post-COVID-19 era was conducted by examining the role of perceived health risk and attitude toward risk in mediating the relationship between tourism knowledge and the coastal ecotourist visit intention.

Based on Theory of Reasoned Action (TRA), the two antecedents of behavioral intention were determined, including attitude and subjective norm. In particular, this research focuses on extending TRA and prior studies to examine the role of perceived health risk and attitude toward risk in mediating the relationship between tourism knowledge and the coastal ecotourist visit intention. The proposed hypotheses were examined using a t test with a 1 percent alpha coefficient. The proposed research was examined by applying empirical data collected from 348 domestic ecotourists in CMC Tiga Warna coastal ecotourism. The results indicated that the validity and reliability test fulfilled all of the criteria, supporting the five hypotheses within the structural equation model, concluding that the results contributed theoretically and in managerial implications.

In analyzing the data, this study had navigated the empirical evidence regarding the significant and direct role of tourism knowledge as a predictor of the coastal ecotourist visit intention, perceived health risk and attitude toward risk in the context of coastal ecotourism. In this study, tourism knowledge particularly proved to be the most effective predictor of the coastal ecotourist visit intention through mediation pathways by perceived health risk and attitude toward risk in separate pathways. Thus, tourism knowledge was regarded as a predictor of the coastal ecotourist visit intention, either directly or indirectly. Tourism knowledge in this study was in the form of knowledge regarding the tourism to be visited, the emergence causes of tourism risks, the consequences of tourism risks and the solutions to tourism risks. Particularly, attitude toward risk was found to have the second largest influence on the coastal ecotourist visit intention. Attitude toward risk in this study included wise actions to comply with health protocols, supportive ideas to comply with health protocols, supportive travel decisions, and cautious actions while complying with health protocols. The visit intention in this study included the indicators such as possibilities, desires, intentions, and travel plans. Additionally, the visit intention was also found to be negatively affected by perceived health risk.

Developing the coastal ecosystem management strategy will require further methodology and data. As a result, the purpose of this research is to identify and comprehend the coastal ecotourist visit intention model, as well as to use the model to aid in the creation of the management strategy. The argument is that empirical conditions exist at the research location where the coastal ecotourism management strategy is carried out by involving the community and ecotourists, as well as management, accommodating stakeholders, and related experts. Visitors describe themselves as stakeholders, which is one of their comments. As an outcome, the coastal ecotourist visit intention would be utilized to provide empirical data to support developing the coastal ecosystem management strategy. In addition to managing conservation and utilization for ecotourism, CMC Tiga Warna manager should work to increase tourist interest, for example, by strengthening tourist information to netizens that CMC Tiga Warna is health friendly and environmentally friendly, reducing health risks, and improving tourist attitudes. As a conclusion, the development of coastal ecosystem management strategy executed at the study site is expected to increase economic, ecological, and social sustainability of coastal ecotourism management.

### 5.1 Effect of tourism knowledge on the level of perceived health risk, attitude toward risk, and coastal ecotourist visit intention to CMC Tiga Warna ecotourism

The results of data analysis indicated that tourism knowledge significantly affected the level of perceived health risk, in which direct attitude toward risk and the visit intention to CMC Tiga Warna coastal ecotourism was −0.171 (p < 0.001), 0.392 (p < 0.001), and 0.205 (p < 0.001), respectively, in which higher tourism knowledge level generates lower perceived health risk level and vice versa. The respective order of tourism knowledge from the largest to the smallest was the effect of tourism knowledge on attitude toward risk, coastal ecotourist visit intention, and perceived health risk, indicating that the role of tourism knowledge was deemed pivotal in determining the level of attitude toward risk. Further, attitude toward risk determines the amount of the visit intention. In addition, the tourism knowledge level of visitors also played an important role in determining perceived health risk. Furthermore, perceived health risk could determine the strength and weakness of the visit intention. Since this study was conducted in the context of coastal ecotourism; hence, the findings of this study extended previous research by Zhu and Deng (2020) regarding the rural tourism intention in China, Cong (2021) regarding the perceived risk and destination knowledge in satisfaction-loyalty intention relationship of European tourists in Vietnam, Utama and Setiawan (2020) in Jabodetabek tourism, Schroeder et al., (2013) about travel intention to London, and Wang and Hazen (2016) regarding purchase intention of remanufactured products in China. The findings of this research additionally developed TRA by adding two factors along with the risk attitude to determine the coastal ecotourist visit intention, which were tourism knowledge and perceived health risk.

According to an empirical study related to profile of tourism knowledge variable, it was found that visitors to CMC Tiga Warna had a significantly adequate tourism knowledge scope, mainly related to information regarding the tourism to be visited, along with the possible risks of travel. The contracting risk of COVID-19 at tourist sites that have implemented procedures and visit restrictions became the most important factor in determining perceived health risk variable. Visitors perceived that it was important to consider the contracting risk of COVID-19 despite the health protocol implementation and restriction in tourist site. During the COVID-19 pandemic, visitors were relatively able to refrain from traveling due to the worrisome of exposing and contracting to COVID-19. This notion was similar to visitors’ assessment that perceived health risk in the form of the exposure risk to COVID-19 remained in the tourist site, thereby requiring cautious actions while implementing health protocols.

The application of health protocols in CMC Tiga Warna ecotourism area was considered “significantly good idea” by visitors, implemented by visitors by complying with health protocols in tourist areas. Consequently, visitors perceived that it was significantly important to comply with health protocols in tourist sites in order to avoid the contracting risk of COVID-19. This finding indicated that CMC Tiga Warna visitors assessed the exposure risk of COVID-19 at a moderate risk level. The behavior of visit intention to CMC Tiga Warna was reflected in the intention and desire to travel, further indicating that visitors who had a strong intention and desire to travel, tended to have a higher level of the visit intention to CMC Tiga Warna. The finding of this study had also emphasized that visitors had a strong interest in traveling to CMC Tiga Warna ecotourism. Based on the discussion, tourism knowledge was proven not only as an antecedent of the coastal ecotourist visit intention, but also to attitude toward risk and perceived health risk.

### 5.2 The role of perceived health risk and attitude toward risk in determining coastal ecotourist visit intention to CMC Tiga Warna ecotourism

The results of data analysis indicated that perceived health risk significantly affected the coastal ecotourist visit intention to CMC Tiga Warna ecotourism directly which was −0.182 (p < 0.001). This result indicated that the role of high and low levels of perceived health risk could affect the coastal ecotourist visit intention level to CMC Tiga Warna ecotourism. Lower level of perceived health risk generates higher level of the visit intention, and vice versa. Further, since this study was conducted in the context of coastal ecotourism, this study extended previously conducted studies (Schroeder et al., 2013; Wang and Hazen, 2016; Wang et al., 2018; Bashir et al., 2019; Utama and Setiawan, 2020; Zhu and Deng, 2020) in travel intention to London, purchase intention of remanufactured products in China, behavioral intention towards green hotel, Jabodetabek tourism, and rural tourism intention in China, respectively.

Data analysis indicated that attitude toward risk significantly affected the visit intention to CMC Tiga Warna ecotourism directly, which was 0.269 (p < 0.001). This result denoted that attitude toward risk determined both high and low level of the visit intention to CMC Tiga Warna ecotourism. Higher level of attitude toward risk generates higher level of the visit intention, and vice versa. In this study, the effect of attitude toward risk on the visit intention was ranked second after the indirect effect. As for the direct effect to the visit intention, attitude toward risk was the biggest factor in determining the visit intention. Through this study, attitude toward risk was also proven to be one of determinants of the visit intention as in TRA (Ajzen and Fishbein, 1980).

### 5.3 The role of perceived health risk and attitude toward risk as a mediating variable between tourism knowledge and coastal ecotourist visit intention to CMC Tiga Warna ecotourism

The results of data analysis indicated that perceived health risk and attitude toward risk mediate the significant positive relationship between tourism knowledge and the visit intention to CMC Tiga Warna ecotourism with an effect of 0.137 (p = 0.005). This result denoted that perceived health risk and attitude toward risk were proven to be effective in acting as mediating variables between tourism knowledge and the visit intention. As for the direct effect of tourism knowledge on the visit intention; along with the indirect effect, perceived health risk and attitude toward risk became the only highest indicator on the visit intention to CMC Tiga Warna ecotourism. The findings of this study provided new empirical evidence in the context of coastal ecotourism, thus this study is the expansion from previous research research by Zhu and Deng (2020) regarding rural tourism intention in China. In general, it is concluded that higher tourism knowledge level demonstrated by visitors, followed by low level of perceived health risk and high level of attitude toward risk, would form stronger the visit intention.

### 5.4 Development of coastal ecotourism management strategy in CMC Tiga Warna, Malang of East Java Province

Coastal ecosystem management as a long-term strategy in CMC Tiga Warna mainly is the result of collaboration between managers as decision maker, scientist, ecotourist, and local community. They make an effort to continuously execute conservation programs and limit their use through ecotourism activities in order to ensure the economic, ecological, and social sustainability of coastal ecotourism management. Interrelationships that exist between multiple, and often competing, parts of the environment as they collaborate to create ecosystem services in CMC Tiga Warna (Abidin et al., 2021). Thus, the sustainable of coastal ecosystem management practices in CMC Tiga Warna have far reaching implications. Integrating the ecological, economic as well as social dimensions of ecosystem services is important to be a reality to make sustainable coastal ecosystem management in CMC Tiga Warna by making a collaborative effort between managers, and scientist such as those seen in Caribbean Small Island Developing States (Ali et al., 2018), ecotourist, and also local community.

Coastal ecosystem management has been implemented in numerous regions in Indonesia, including a strategy for increasing institutional collaboration between villages and communities on mangrove ecosystem management in Lembar Bay, Lombok (Sukuryadi et al., 2021), mangrove ecotourism management in the Special Region of Yogyakarta, especially for educational purposes (Khakhim et al., 2021), utilizing social-cultural values in constructing conservation character education in the management of coastal biodiversity in Gorontalo Province (Katili et al., 2018) and coastal ecotourism management using a Community-Based Tourism (CBT) model in Panggul, Trenggalek of East Java Province (Sarinastiti and Wicaksono 2021). This CBT model has already been employed in CMC Tiga Warna for the management of coastal and sea ecosystems, and has even involved tourist visitors in conservation-based ecotourism activities (Abidin et al., 2021).

On the basis of the readiness of the local community in preparing and implementing health protocols in tourist areas, as well as with permission from the Malang Regency tourism office, CMC Tiga Warna ecotourism has opened tourism services in the COVID-19 pandemic period since August 8, 2020 while consistently complying with applicable rules. Changes in tourist behavior in the pandemic period are essential, such as seen in the visit intention to CMC Tiga Warna, serving to be consider as supporting for the future coastal ecotourism management strategy. According to Ali et al., (2018), coastal ecosystem management needs to involve decision makers and scientists. The alternative formulation of development strategy for coastal ecotourism management in this study incorporates not only managers as decision maker, but also scientist, visitors, and local community through conservation and ecotourism activities. They were invited in the context of environmental education. Visitors in this study were questioned by the visit intention's linked answer that affiliated with tourism knowledge, perceived health risk, attitude toward risk, and the results (Table 6) revealed that the visit intention was more dominantly influenced by tourism knowledge. This means that an increase in tourism knowledge is achieved in relation to conservative CMC Tiga Warna coastal ecotourism information and the health-conscious guest. This will boost the visit intention to CMC Tiga Warna, lower level of perceived health risk, and strengthen attitude toward risk. The increase in the visit intention has an impact on the potential for increasing the number of tourist visits, so the sustainability of the economic incentives received by managers and the community also increases as a motivation and capital to maintain the conservation program as the development of coastal ecotourism management strategy. As a result, the coastal ecotourist visit intention was discovered to be a proponent of development of coastal ecotourism management strategy in CMC Tiga Warna. For this reason, this study aims to utilize research findings related to the behavior of visit intention during the post COVID pandemic to become one of supporting part for strengthening the development of coastal ecotourism management strategy in welcoming the tourists in the post-pandemic-COVID-19 period and in the new normal era.

Based on research findings regarding the visit intention model, strengthening the coastal ecotourism management strategy through conservation program was supported by increasing the visit intention to CMC Tiga Warna. Such as seen in 2019, increasing level in the visit intention encouraged the recovery of the tourist numbers as which managed to reach the specified carrying capacity. At that time, the annual average visit reached 58,000 visitors, with a total economic benefit of IDR 2.7 billion. The recovery of CMC Tiga Warna ecotourism visit carrying-capacity could certainly provide economic incentives for local communities, as well as support the development strategy of coastal ecotourism management, thereby encouraging ecological and social incentives in a sustainable manner.

The increasing level in the visit intention to CMC Tiga Warna ecotourism in the post-COVID-19 era was achieved by providing adequate tourist information, required by prospective tourists on the official CMC Tiga Warna website and social media; in which the attempts include: updates on weather conditions, tides, potential tsunami, various tourist objects such as coastal areas, anticipated potential risks when traveling, specific type of luggage for visitors when visiting CMC Tiga Warna, cautious emergence of travel risks, as well as consequences and solutions for these risks. In addition, CMC Tiga Warna is suggested to consistently minimize the exposure risk of COVID-19 while enforcing health protocols in tourist areas, to implement the carrying capacity as part of efforts to maintain the distance between visitors to avoid crowds, to process the ownership and application of CHSE (Cleanliness, Health, Safety, and Environment) certification at tourist sites, as well as to improve the availability of health facilities and infrastructure. In addition, it is deemed pivotal to strengthen attitude toward risk by convincing netizens and visitors that CMC Tiga Warna ecotourism is safe for visit in the post-COVID-19 era, as it continues to consistently implement health protocols in tourist areas. CMC Tiga Warna managers are also advised to increase visitors' understanding regarding health risks in the post-COVID-19 era during their trip; thus, they are ready to avoid crowds, to constantly wear masks, to maintain a physical distance, and to comply with other health protocols.

## 6 Conclusions, limitations and implications

This study introduces a novel concept: the use of coastal ecotourist visit intention as one of the predictors of visit decision to the tourism destination in the context of marketing management, as a supporting component in developing the coastal ecosystem management strategy in CMC Tiga Warna ecotourism. The strategy should be implemented in accordance with conservation principles at all times, engaging the role of ecotourists and the local community, and considering the visit intention model as a supporting part of developing the strategy, while restricting the utilization of coastal ecosystems through ecotourism. Visit intention to CMC Tiga Warna is positively affected by tourism knowledge and risk attitude. However, perceived health risk has a negative relationship. The most effective pathway for increasing the visit intention in the post-COVID-19 period was found to be perceived health risk and attitude toward risk mediated tourism knowledge. As a result, the coastal ecosystem management development strategy in CMC Tiga Warna could be helped by increasing the visit intention in order to provide economic incentives within the carrying capacity limit, as well as by supporting a sustainable coastal ecotourism management strategy, thereby encouraging ecological and social incentives in a sustainable manner. This study's findings also contributed to the development of the Theory of Reasoned Action by incorporating two additional factors, tourism knowledge and perceived health risk, in addition to risk attitude, to determine the visit intention. Finally, CMC Tiga Warna managers are encouraged to actively provide various tourist information and knowledge as requested by prospective visitors, as well as to remain consistent in minimizing COVID-19 exposure risk at tourist sites and strengthening attitude toward risk to improve the visit intention.

Cross-sectional studies, such as the one used in this study during the Covid-19 pandemic, are ineffective for determining cause-and-effect relationships. As a consequence, it simply describes a direction and correlational relationship, as in this study. The purpose of this study is to describe the relationship between tourism knowledge and visit intention behavior during COVID-19 pandemic outbreaks, as mediated by perceived health risk and risk attitude. As a result, this finding is more effective in explaining coastal ecotourist visit intention during Pandemic Covid-19 with tourism knowledge as an antecedent of visit intention, instead of through an indirect relationship through the use of perceived health risk and attitude toward risk. However, changes in visitors’ behavior for almost two years Pandemic Covid-19, for example, in the case of the visit intention behavior are influenced by tourism knowledge, perceived health risk, and attitude toward risk, should be a concern of CMC Tiga Warna tourism managers by utilizing this research finding. This study is also unique because it focuses on coastal ecotourism management at the CMC Tiga Warna in Malang, which is located in the East Java Province. Consequently, the research findings could be utilized by those who manage ecosystems containing similar types of things. CMC Tiga Warna differentiates itself by ecotourism in coastal areas controlled by conservation-based communities, by including local communities into the Community-Based Tourism (CBT) concept, and by engaging travelers in conservation activities. The findings to implement the visit intention model on the coastal ecosystem management development strategy are also novel ideas, as the management of coastal resources requires the participation of coastal ecotourism-related stakeholders such as managers, experts, local communities, visitors, community group supervisors, governments, and multidisciplinary scientists. However, the findings of the coastal ecosystem management strategy model developed at the site of this study are mainly based solely on the viewpoints of visitors, local communities, and managers; therefore, this concept requires future research that incorporates additional components in the construction of coastal ecosystem management strategies through conservation and ecotourism.

## Declarations

### Author contribution statement

Zainal Abidin: conceived and designed the research; performed the research; analyzed and interpreted the data; wrote the paper.

Wahyu Handayani: conceived and designed the research; performed the research; analyzed and interpreted the data; wrote the paper.

Emeraldo A. Zaky: Contributed reagents, materials, analysis tools or data; analyzed and interpreted the data.

Achmad D. Faturrahman: Contributed reagents, materials, analysis tools or data; analyzed and interpreted the data.

### Funding statement

Mr. Zainal Abidin was supported by Universitas Brawijaya [DIPA-023.17.2.677512/2021].

### Data availability statement

Data included in article/supp. material/referenced in article.

### Declaration of interest’s statement

The authors declare no conflict of interest.

### Additional information

No additional information is available for this paper.
